# Association between Downstream Taste Signaling Genes, Oral Microbiome, and Severe Early Childhood Caries

**DOI:** 10.3390/ijms24010081

**Published:** 2022-12-21

**Authors:** Vivianne Cruz de Jesus, Betty-Anne Mittermuller, Pingzhao Hu, Robert J. Schroth, Prashen Chelikani

**Affiliations:** 1 Manitoba Chemosensory Biology Research Group, Department of Oral Biology, University of Manitoba, Winnipeg, MB R3E 0W2, Canada; 2 Children’s Hospital Research Institute of Manitoba (CHRIM), Winnipeg, MB R3E 3P4, Canada; 3 Department of Preventive Dental Science, University of Manitoba, Winnipeg, MB R3E 0W2, Canada; 4 Department of Biochemistry and Medical Genetics, University of Manitoba, Winnipeg, MB R3E 3N4, Canada; 5 Department of Biochemistry, Western University, London, ON N6A 5C1, Canada; 6 Department of Pediatrics and Child Health, University of Manitoba, Winnipeg, MB R3A 1S1, Canada; 7 Department of Physiology and Pathophysiology, University of Manitoba, Winnipeg, MB R3E 0J9, Canada

**Keywords:** dental caries, tooth decay, bacteria, fungi, taste receptors

## Abstract

Polymorphisms in taste receptor genes have been shown to play a role in early childhood caries (ECC), a multifactorial, biofilm-mediated disease. This study aimed to evaluate associations between severe-ECC (S-ECC), the oral microbiome, and variants in genes that encode components of the G protein-coupled receptor (GPCR) signaling cascade involved in taste sensation. A total of 176 children (88 caries-free; 88 with S-ECC) were recruited. Analyses of *16S* and *ITS1 rRNA* microbial genes and seven (*GNAQ, GNAS, GNAT3, GNAI2, RAC1, RALB*, and *PLCB2*) human genes were pursued using next-generation sequencing. Regression analyses were performed to evaluate associations between genetic variants, S-ECC, and the supragingival plaque microbiome. Results suggest that *PLCB2* rs2305645 (T), rs1869901 (G), and rs2305649 (G) alleles had a protective effect on S-ECC (rs2305645, odds ratio (OR) = 0.27 (95% confidence interval (CI): 0.14–0.51); rs1869901, OR = 0.34 (95% CI: 0.20–0.58); and rs2305649, OR = 0.43 (95% CI: 0.26–0.71)). Variants in *GNAQ, GNAS, GNAT3, PLCB2, RALB*, and *RAC1* were associated with oral fungal and bacterial community composition. This study revealed that three loci at *PLCB2* are significantly associated with S-ECC. Variants in multiple genes were associated with the composition of dental biofilm. These findings contribute to the current knowledge about the role of genetics in S-ECC.

## 1. Introduction

Most taste modalities are sensed through G protein-coupled receptors (GPCRs). They represent the largest receptor superfamily in humans and are composed of seven canonical transmembrane-spanning proteins [1]. GPCRs can elicit cellular and physiological responses by converting external stimuli into intracellular signals by recognizing and binding sensory input and ligands, including odors, tastes, bioamines, lipids, peptides, and proteins [1]. Chemosensory receptors such as odorant and taste receptors account for over half of the GPCR superfamily. Several studies have highlighted the diverse roles of these chemosensory receptors and have been extensively reviewed [2,3,4,5].

The signal transduction of GPCRs involves different components such as cell surface receptors, GTP-binding proteins (G proteins), second messengers, and effector molecules. GPCR-mediated calcium (Ca^2+^) signaling is essential for several physiological functions and dysregulations in this process are associated with many diseases, such as Alzheimer’s, Huntington’s disease, and Parkinson’s disease [6]. In a previous study, we showed that GPCRs and other proteins involved in detecting different taste signals, such as epithelial sodium channels, otopetrin 1, and carbonic anhydrase 6, are associated with severe early childhood caries (S-ECC), and the oral microbial composition of young children [7]. Here, we tested the hypothesis that variants in downstream components of the G protein-mediated GPCR signaling cascade may have similar effects.

Variants in genes that encode components of the GPCR signaling cascade may interfere with the host-microbial interactions mediated by taste receptors, as well as with taste preferences and dietary choices, leading to increased risk of S-ECC. Furthermore, Ca^2+^ is a well-known intracellular second messenger involved in GPCR signaling. In the context of oral health, Ca^2+^ signaling is important for tooth formation and components of the GPCR signaling cascade may be involved in the development of tooth and bone defects [8,9,10]. For instance, a study showed that increased Gαq activity in ameloblasts is linked to dental fluorosis, a tooth defect caused by increased ingestion of fluoride during enamel formation [11]. Activity of another G protein, Gαs, has been shown to modulate bone health and development [12]. Sweet taste receptors T1R2 and T1R3 have been associated with modulation of bone development, with reports of T1R2 and T1R3 knockout mice showing increased bone mass [13]. Additionally, defects in free fatty acid receptors FFAR1 and FFAR4 have been implicated in decreased bone formation and increased bone resorption in mice models [8]. As there are similarities in the process of bone and tooth formation, proteins involved in bone defects may also influence the formation of tooth defects [14]. Furthermore, tooth defects, along with dietary choices, which can be determined by taste preferences mediated by taste receptors, are known risk factors for early childhood caries (ECC) [15,16,17,18].

The canonical bitter taste signaling cascade mediated by heterotrimeric G-proteins suggests that the activation of taste receptors by a ligand leads to the activation of the intracellular heterotrimeric G-protein complex, Gαβγ. Then, the Gβγ dimer activates phospholipase C β2 (PLCβ2), which leads to Ca^2+^ release from the endoplasmic reticulum. Meanwhile, the Gαgustducin subunit activates phosphodiesterase to decrease cyclic AMP (cAMP) levels [2,4,19,20,21]. Studies have suggested that multiple Gα subunits, such as Gα_i_, Gα_s_, and Gα_q_, expressed in taste buds may also be involved in taste receptor signaling [22,23].

Upon activation of bitter taste receptors (T2Rs), the released second messenger Ca^2+^ binds to Ca^2+^-binding proteins, such as calmodulin, to continue the calcium-dependent signal transduction. Research has shown that calmodulin can interact with small GTPases such as Rac1 and RalB, suggesting that they may be involved in many calcium/calmodulin-mediated intracellular signaling pathways [24,25]. Recently, it was shown that Rac1 GTPase activity was regulated by quinine with the signal mediated by G-protein and T2R4 [26].

The objective of this study was to analyze the association between variants in downstream taste signaling genes, the oral microbiome, and S-ECC. While there are dozens of downstream proteins that might be potentially involved in human taste signaling, based on the previous published literature and to keep this objective feasible, the focus was only on seven downstream proteins. Thus, associations between variants in genes encoding cell signaling components, such as heterotrimeric G proteins (*GNAT3, GNAS, GNAI2*, and *GNAQ*), a downstream effector (*PLCB2*), and small GTPases (*RAC1* and *RALB*) were assessed. The findings suggest that variants in the *PLCB2* gene are significantly associated with S-ECC and that genetic variants of other signaling components are associated with the composition of the oral microbiota.

## 2. Results

### 2.1. Association between Genetic Variants and S-ECC

Eighty-eight caries-free children (45.34 ± 14.69 months old, 43 females, 45 males) and 88 children with S-ECC (44.88 ± 11.84 months old, 51 females, 37 males) were recruited. No significant differences in age and sex were identified between the groups (*p* > 0.05).

**Figure 1:**
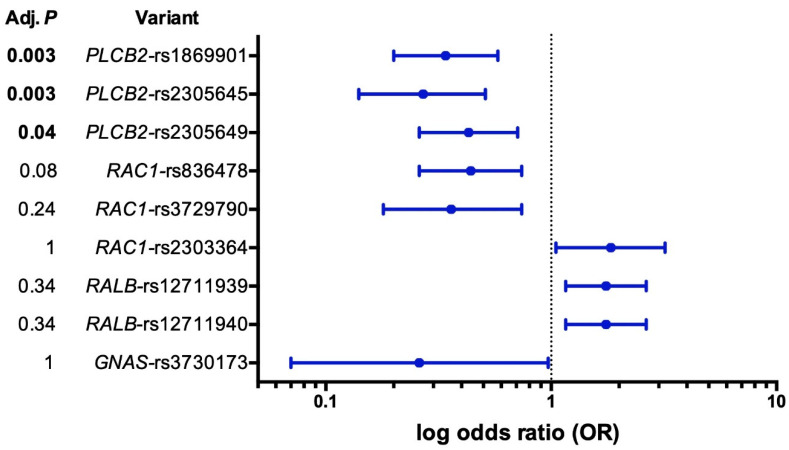
Forest plot for the results of logistic regression. Effect sizes are expressed on a log OR scale. Adj. *p*, *p* corrected for multiple testing by Bonferroni adjustment. Adjusted *p* < 0.05 were considered statistically significant.

After correcting for multiple testing, the case–control allelic association analysis showed that three variants in *PLCB2* were significantly associated with S-ECC (adj. *p* < 0.05, Table 1). Logistic regression analysis, adjusting for sex and age, showed that children with *PLCB2* single nucleotide polymorphisms (SNPs) rs2305645 (T, OR = 0.27, 95% CI = 0.14–0.51), rs1869901 (G, OR = 0.34, 95% CI = 0.20–0.58), and rs2305649 (G, OR = 0.43, 95% CI = 0.26–0.71) had lower odds of developing S-ECC (i.e., they are protective SNPs; adj. *p* < 0.05, Figure 1).

### 2.2. Association between Bacterial, Fungal Microbiota and Host Genetic Variants

The results of the differential abundance analysis between the S-ECC and caries-free groups have been published previously [7]. Results from the linear regression model showed that eleven variants were positively or negatively correlated with the abundance of bacterial or fungal taxa. No significant associations were observed between the host variants and alpha (within samples) Shannon diversity (Adj. *p* > 0.05). None of the three *PLCB2* SNPs associated with S-ECC significantly correlated with microbial species after adjustment for multiple testing. The significant associations are shown in Table 2.

## 3. Discussion

This work builds on the previous study assessing the role of variants in genes encoding taste receptors, ion channels, and the oral microbiome in S-ECC risk/protection [7]. It was previously identified that polymorphisms in taste-related genes are associated with S-ECC and that raised the question of whether variants in genes encoding downstream taste signaling components and other proteins involved in GPCR signaling could also play a role.

PLCβ2 is a critical component of the taste signaling cascade. A recent study looked at the expression of PLCβ2 in oral cells, using published data from single-cell RNA-seq studies, and reported that PLCβ2 was expressed in almost all endothelial, epithelial, fibroblast, and immune cell subsets evaluated [5]. PLCβ2 is responsible for the generation of the second messenger inositol-1,4,5—triphosphate (IP_3_), which activates IP_3_ receptors in the endoplasmic reticulum (ER), releasing Ca^2+^ into the cytoplasm [27]. Therefore, mutations in *PLCB2* can have an important effect on Ca^2+^-mediated physiological functions. The findings from this study suggest that *PLCB2* rs2305645 (T), rs1869901 (G), and rs2305649 (G) are protective SNPs against S-ECC. Interestingly, rs1869901 has been associated with autism spectrum disorder [28].

In this study, the association between mutations in downstream taste signaling components and the oral fungal and bacterial community composition was also evaluated. The results showed that variants in *GNAQ, GNAS, GNAT3, RAC1, RALB*, and *PLCB2* were correlated with the relative abundances of bacterial taxa, while two *RAC1* SNPs were correlated with the relative abundance of fungi. Although none of the three *PLCB2* variants that were associated with S-ECC were correlated with oral microbial composition, another *PLCB2* variant (rs72731486) was negatively associated with an unclassified *Streptococcus* species. An unclassified fungus from the order *Malasseziales* was previously associated with healthy dental plaque [7] and here it was shown to be negatively correlated with the *RAC1* rs3729790 variant.

Though the canonical taste signaling pathway suggests that taste receptors activate Gαgustducin, the co-expression of multiple Gα subunits in taste buds have suggested that other Gα subunits may be involved in taste sensation [22]. Furthermore, recent studies suggest that taste receptors are involved in multiple functions, unrelated to taste sensation, in which other Gα subunits may be involved. For instance, a study showed that the T2Rs expressed in human airway smooth muscle, where their activation leads to relaxation and bronchodilation, couple to Gαi instead of Gαgustducin [29]. Therefore, it is possible that variants in the genes encoding Gα subunits may affect the taste receptor signal transduction and other aspects of oral health.

Variants in small G proteins may affect receptors that mediate microbial recognition. RalB is a small GTPase, a member of the Ras GTPase superfamily. It is activated by calcium/calmodulin interactions and is involved in cytoskeleton rearrangement and vesicle trafficking. RalB has been implicated in several human cancers such as oral squamous cell carcinoma [30]. Interestingly, RalB has been shown to play a role in innate immunity, helping to trigger innate immune pathways after activation of Toll-like receptors (TLRs) by viruses in human epithelial cells [31]. Furthermore, RalB activation after microbial stimuli in macrophages can induce autophagy, a key process for the clearance of intracellular pathogens [32]. This agrees with our finding that *RALB* variants may be associated with the composition of the oral microbiome. Rac1 (Ras-related C3 botulinum toxin substrate 1) is also a small G protein and it belongs to the family of Rho GTPases. It plays a role in various cellular functions such as actin cytoskeletal reorganization, cell cycle regulation, and movement [26,33]. Rac1 is activated by a variety of receptors, including GPCRs. Recent studies from our group showed that there is a link between Rac1 and T2Rs [26,34]. This link may involve the role of Rac1 in actin cytoskeletal reorganization and T2R-mediated internalization of Gram-positive bacteria, as well as the T2R4- and G protein-dependent inhibitory effect of Rac1 activity by the known T2R agonist quinine [26,34]. It is possible that variants in *RAC1* could affect the T2R-mediated internalization of microbes, which would justify the correlation of *RAC1* variants with the abundance of oral bacteria and fungi.

It is important to note that this study is not free of limitations. There was no information about some of the potentially confounding variables for the association observed, such as lifestyle and socioeconomic status. Despite having a small sample size, significant associations were detected. This is the first study to suggest an association between mutations in *PLCB2* and decreased odds of S-ECC. Further work is needed to characterize this association and to investigate the correlations between variants in genes encoding G proteins and the composition of bacterial and fungal communities in dental plaque.

## 4. Materials and Methods

### 4.1. Study Design

A total of 176 children younger than 72 months of age with S-ECC or caries-free controls were recruited in Winnipeg, MB, Canada. The inclusion criteria were children younger than 72 months of age; child has S-ECC (American Academy of Pediatric Dentistry definition) [35], or is caries-free (dmft index = 0, i.e., no decayed, missing, or filled primary tooth surface; no incipient lesions). The exclusion criteria were age (>72 months); child with caries but who does not satisfy the case definition of S-ECC; current use of antibiotics. Further details about the study population and methods used for the evaluation of variants in candidate genes and association between host variants and the oral microbiome have been described previously [7].

Supragingival plaque and oral swab samples were collected for the study of the plaque microbiome (bacteriome and mycobiome) and the genetic variants in the seven genes involved in taste and calcium signaling, respectively (Table 3). The samples were stored in RNAprotect solution (Qiagen, Hilden, Germany) and kept at −80 °C until further analysis.

Written informed consent was obtained from all parents or legal caregivers and the study protocol was approved by the University of Manitoba’s Health Research Ethics Board (HREB HS23754-393 H2020:150) and the Misericordia Health Centre.

### 4.2. DNA Sequencing and Data Analysis

The paired-end targeted V4-*16S rRNA* and *ITS1 rRNA* amplicon sequencing (MiSeq PE250, Illumina Inc., San Diego, CA, USA) was performed by Génome Québec Innovation Centre (Montréal, Canada), using DNA extracted from supragingival plaque samples. The data were analyzed using the QIIME2 (v2018.11) pipeline [36] and various R packages (“qiime2R” v0.99.13, “phyloseq” v1.30.0, “DESeq2” v1.26.0), as previously described [7]. The negative binomial Wald test implemented in the “DESeq2” R package [37] was used for differential abundance analysis, controlling the false discovery rate (FDR) for multiple comparisons, and adjusting for sex and batch effect (library preparation, sequencing runs, DNA extraction batches). The Shannon diversity index was calculated using the R package “phyloseq” (version 1.30.0). It was used to detect associations between host genetics and microbial diversity.

The paired-end targeted sequencing of the seven genes (Table 3), using DNA extracted from oral swabs, was performed by Génome Québec Innovation Centre (Montréal, Canada). The Fluidigm Access Array technology (Fluidigm, South San Francisco, CA, USA) was used for library preparation and the NovaSeq6000 SP PE250 (Illumina Inc., San Diego, CA, USA) platform was used for sequencing. The primers used are listed in Table S1.

The genetic sequence data were analyzed using the GATK (v4.2.0, Broad Institute) best practices pipeline, Picardtools (v2.25.0), VCFtools (v0.1.15), and PLINK (v1.9) [38,39,40]. A total of 662 genetic variants (SNPs and INDELS) were called. Variants with >5% missing genotype call rate, mean sequencing depth < 10x, significantly different (*p* < 0.00001) missing data rate between cases and controls, Hardy–Weinberg equilibrium (HWE) *p*  <  0.05 in controls, and MAF < 0.01 were removed. Samples with a genotype failure rate > 0.5 were also removed. After quality control, 53 variants and 174 samples were included in downstream analyses. The filtered variants were then annotated using SnpEff 5.0e [41]. A case–control allelic association analysis was used to evaluate the association between host genetic variants and S-ECC using PLINK. The allelic chi-squared (χ^2^) test was used to identify significant differences in allele frequencies. Regression models were further used to analyze the associations between host genetic variants and S-ECC, and host genetic variants and the oral microbiome with an additive genetic model using PLINK, adjusting for age, sex, and microbiome sequencing batch [7].

## Figures and Tables

**Table 1:** Allelic association of genetic variants with severe early childhood caries (S-ECC).

Gene	Variants	Location (GRCh38)	Effect Allele	Frequency of Effect Allele		χ^2^	*p*	OR	Adj. p *	Type of Variant
				Cases (S-ECC)	Controls (Caries-Free)					
*PLCB2*	rs2305645	chr15: 40303364	T	0.09	0.27	19.05	1.28 × 10^−5^	0.27	0.0007	Intron
	rs1869901	chr15: 40303426	G	0.17	0.38	18.43	1.77 × 10^−5^	0.34	0.0009	Intron
	rs2305649	chr15: 40297629	G	0.16	0.34	13.96	0.0002	0.38	0.0099	Intron

Note: * Adjusted *p* (corrected for multiple testing by Bonferroni adjustment test) less than 0.05 were considered statistically significant. Only significant associations are shown. χ^2^, basic allelic test Chi-square. The basic allelic test compares frequencies of alleles in cases versus controls. Chr, chromosome. OR, odds ratio.

**Table 2:** Association between host genetic variants and oral bacterial and fungal taxa.

Gene	Variant	Location	Allele	BETA	Adj. *p*	Taxa
**BACTERIA (*n* = 174)**						
*GNAQ*	c.736-12T > C	chr9:77728679	G	−1.241	0.0029	*Capnocytophaga sputigena*
*GNAQ*	rs1478186975	chr9:77728678	G	−2.192	0.0311	*Actinomyces gerencseriae*
*RAC1*	rs1051504128	chr7:6387120	A	6.913	0.0095	Unclassified bacteria
*RAC1*	rs836478	chr7:6392059	C	0.534	0.0443	*Lachnospiraceae [G-3] bacterium* HMT 100
*GNAS*	rs3730173	chr20:58909879	T	−3.14	0.0208	*Lachnoanaerobaculum saburreum*
*RALB*	c.356A > G	chr2:120289612	G	−3.14	0.0208	*Lachnoanaerobaculum saburreum*
*RALB*	rs11545293	chr2:120278757	A	0.7232	0.0311	*Bergeyella sp.* HMT 907
*PLCB2*	rs72731486	chr15:40299054	A	−0.5803	0.0325	Genus *Streptococcus*
*GNAT3*	rs6975345	chr7:80494683	C	2.755	0.0351	*Prevotella salivae*
**FUNGI (*n* = 155)**						
*RAC1*	rs836478	chr7:6392059	C	2.519	0.0324	Genus *Alternaria*
*RAC1*	rs3729790	chr7:6387323	A	−2.494	0.0329	Order *Malasseziales*

Beta, regression coefficient. Adj. *p* < 0.05 are corrected for multiple testing by Bonferroni adjustment test.

**Table 3:** List of sequenced genes.

Gene Symbol	Gene Name	RefSeq ID	Location	Function
*GNAS*	G protein subunit alpha s	NM_001077489	Chr20: 58,891,364–58,911,192	It encodes the guanine nucleotide-binding protein Gαs, which is involved in activation of adenylyl cyclase (AC), the enzyme that synthesizes cyclic adenosine monophosphate (cAMP) from adenosine triphosphate (ATP), and a variety of cellular responses. With relevance to dental and oral health, mutations in this gene are linked to bone defects.
*GNAI2*	G protein subunit alpha i2	NM_002070	Chr3: 50,236,204–50,259,362	It encodes the Gαi2 subunit, which is involved in hormonal regulation of AC. There is evidence of interaction between Gαi2 and bitter taste receptors (T2Rs).
*GNAQ*	G protein subunit alpha q	NM_002072	Chr9: 77,716,097–78,031,811	It encodes the Gαq subunit, which couples GPCRs and PLCβ.
*GNAT3*	G protein subunit alpha transducin 3	NM_001102386	Chr7: 80,458,635–80,512,064	The Gαgustducin encoded by this gene binds to taste receptors and is involved in the canonical taste signaling pathway.
*PLCB2*	Phospholipase C beta 2	NM_004573	Chr15: 40,287,909–40,307,935	It encodes PLCβ2, which catalyzes the hydrolysis of PIP_2_ to IP_3_, which elicits Ca^2+^ release from internal stores. PLCβ2 is involved in the canonical taste signaling pathway.
*RAC1*	Rac family small GTPase 1	NM_006908	Chr7: 6,374,527–6,403,967	It encodes a GTPase belonging to the RAS superfamily of small G proteins. Members of this superfamily have been shown to regulate a broad number of cellular events such as cytoskeletal dynamics and have a possible link with T2Rs.
*RALB*	RAS-like proto-oncogene B	NM_002881	Chr2: 120,252,852–120,294,710	It encodes a GTP-binding protein that is a member of the small GTPase superfamily and Ras family of proteins. It is involved in innate immunity and tumor growth.

Note: The starting and ending nucleotide positions are from the December 2013 (GRCh38/hg38) human assembly (http://genome.ucsc.edu (accessed on 14 March 2022)).

## Data Availability

De-identified raw *16S* and *ITS1 rRNA* gene sequences derived from the supragingival plaque samples are deposited at NCBI Sequence Read Archive (SRA) Repository (accession number PRJNA555320).

## Supplementary Materials

The supporting information can be downloaded at: https://www.mdpi.com/article/10.3390/ijms24010081/s1.
