# Supplementary material for: Association between Downstream Taste Signaling Genes, Oral Microbiome, and Severe Early Childhood Caries
Source: Int J Mol Sci. 2022 Dec 21;24(1):81. doi: 10.3390/ijms24010081 (PMC9820665; doi:10.3390/ijms24010081)
Supplement: Supplementary file 1 [file ijms-24-00081-s001.zip › ijms-2080981-supplementary.pdf]

Association among downstream taste signaling genes, oral microbiome, and severe early childhood caries

Vivianne Cruz de Jesus[1,2], Betty-Anne Mittermuller[1,2,3],

Pingzhao Hu[2,4,5], Robert J. Schroth[1,2,3,6], Prashen Chelikani[1,2,4,7*]

[1] Manitoba Chemosensory Biology Research Group, Department of Oral Biology, University of Manitoba, Winnipeg, MB, Canada.

[2] Children's Hospital Research Institute of Manitoba (CHRIM), Winnipeg, MB, Canada.

[3] Department of Preventive Dental Science, University of Manitoba, Winnipeg, MB, Canada.

[4] Department of Biochemistry and Medical Genetics, University of Manitoba, Winnipeg, MB, Canada.

[5] Department of Biochemistry, Western University, London, ON, Canada.

[6] Department of Pediatrics and Child Health, University of Manitoba, Winnipeg, MB, Canada

[7] Department of Physiology and Pathophysiology, University of Manitoba, Winnipeg, MB, Canada.

Table S1. List of primers used.

Gene	Forward	Reverse	Amplicon length
GNAI2_1	AATCAAACTCAGCAAGTCATCCG	CTCTTGCAACTAGCCCTCTGTG	341
GNAI2_2	CGCAGTCGCTCGGAACT	GTCAAAGTCCTTGTCTGGGGATT	318
GNAI2_3	GGAACCACCACAGCTCTGAAT	CCTTTCCTGCCCTTTCTCTGG	316
GNAI2_4	CCATGTCCAGAAAGCTGAAGTGT	GGCCACAGTGGTCAAGGTAA	346
GNAI2_5	GCTGGTGGGAAGGTTAGTTCTG	GACCTGCTTCAGAGAAGGGAAAA	336
GNAI2_6	TTCTGAGAAGCAGAAGGACCC	GAAACCTTTTGGGAGGTGGGAGA	329
GNAI2_7	GGTACATTCCTTCAACTGCCTGA	AGGCCTTGGTTAACAGTCATCG	340
GNAI2_8	CAGGGTCCAGCTAAGGAAGC	GTTCACTAGGTGGTGAGGATGTG	340
GNAI2_9	ACCTGAGAAATGGGGTAGAAAGC	GTGAACCACTTGTTGTTGCAGAT	342
GNAI2_10	CTATGACTTGGTGCTAGCTGAGG	CTACTTGGGACAAGAAGCAACCC	326
GNAI2_11	CCTTGCTGCACACGTAGGAT	CTTAGTTCTTCCCCAGCACCC	329
GNAQ_1	CAGAGTCCAGGACGGCAATAAAT	AAAGTGTCCCTCTTGTCTCTTGG	342
GNAQ_2	TGGGGTTTGGAGACAAAACCTAT	CCAACACAGGGCTACAAAAACTG	342
GNAQ_3	ACTCCATTCCCCACACCCTA	ATTTTCCCTAAGTTTGTAAGTAGTGC	308
GNAQ_4	GTACTCAAGGCATAAAAGCTGGG	TTTCCACAGACTCCTCTACCACT	326
GNAQ_5	CCTCCACATGGAAGTAAAGAGAA	AGACCAGTATATGAAGGAAGGTGTT	322
GNAQ_6	TATTTCCAAACCCCCCTATGCAC	TGTGGTCTGATGAGCTGCTATTG	334
GNAQ_7	CAAGGATGGTGGGCTGGG	TGTGTGCGCGCTGTGAG	250
GNAS_1	CTGCAGAGCCAGAGGG	AGGGACAGCTCAAGGTCT	337
GNAS_2	TCCGGCGCCCAGGTAT	TCTGGGGCTCCTGAGTG	349
GNAS_3	GCCCAAGCACTCCACCTTC	GTGGGCAACTAACCTGAATCCAT	347
GNAS_4	CGAAGAGATGGAGACCGAAC	TTCTGGTGGAGGGCTGTAT	340
GNAS_5	AGCCTGGATTCCCCAGT	CCCCAAATCCTTCTCCATCAAG	345
GNAS_6	TCCCAGACCTTGCTCCA	CCCGTGAAGTTAGAAGGAGG	349
GNAS_7	GCCTCCCCTCTGGGTC	GAGAGGGTACTTTTCCTCCCT	311
GNAS_8	GAAGCAGCAGAGATGGAAGGAG	GCATCTGGATCGGCTGGG	332
GNAS_9	ATTCGCAGCCGATCCC	GATGGATCTTGCGTCTGG	311
GNAS_10	CAGGGGCTCCCACTGC	ATCCGGAGGTCCCATCG	282
GNAS_11	GGCCAGACGCAAGATCCAT	GGGACTCTCAGATCGACCGAA	316
GNAS_12	CAACTTACTCCGCAACTTTCTC	CCCCTAGCCCCACCAG	339
GNAS_13	TCCTTGCCGAGGAGCC	CGTGGCCCGGTAGACCT	251
GNAS_14	GTGTTTGGTTTTTTGCATGTTGCT	AGAGCCCTTCCCAGGATTTTCTA	341
GNAS_15	CAGAAAGGCGACCTAAGAATTGC	TGTATGCCAATATGGCTGATGGT	340
GNAS_16	GTGGTGGAGTGGCTATTTCTCAT	TCATCGTTTAAGGTCAGGTAGCA	328

GNAS_17	CTCCTAACTGACATGGTGCAATA	GGGGCTAAGGCCACACAAG	350
GNAS_18	CAAGTGTCGGTCACATAGGGAAC	TAGAACTTTCTGCCAGTGGGGTA	348
GNAS_19	CAAATTGATGTGAGCGCTGTGAA	ACAGTTGGCTTACTGGAAGTTGA	327
GNAS_20	TGGCTTTGGTGAGATCCATTGAC	CCCTAACAACACAGAAGCAAAGC	327
GNAS_21	CTCTGGAATAACCAGCTGTCCTC	TGGGCGGTCACTCCAC	338
GNAS_22	AACAACAGGTTTGTGGAGTGACC	AAAAGAACCACCGCAATGAACAG	334
GNAS_23	GAGAAGCAAGAAAAACGCACTCC	ACACTGGATGTGCGTGAACTAAA	339
GNAS_24	TATGACATCAGAGGCTGGCTGAC	CCTATGGTGGGTGATTAACTGCT	346
GNASE01_1	CAGCGCAACGAGGAGAAG	AGACAGAGCCCGCGAAC	100
GNAT3_1	ACTAAACACGTCATACAGCACCT	GCAGGAAACTACATCAAGAACCAG	250
GNAT3_2	TGATTAGAAAAGCCCACAGTCTT	TGACCGTTGCCTTTATGATTTTGC	327
GNAT3_3	CCAGTTTGGATCATAAAGATAACTCA	AATCCTACCCATTCTCATTTCTG	324
GNAT3_4	TGTTGAGCATCATGAACAAGGAT	CCTCAATGCACATTTTGGAGGAT	328
GNAT3_5	GGCTAGAGCTTTTCTCCATGAGG	CCTGGTTTCTTCAAACGCAAAGT	348
GNAT3_6	AATCTCATTATGTCACCTTGTTC	CTTTATGCAATGGCAAATACCCT	322
GNAT3_7	GCTGCTGAGTCATTGAGCTGATA	GGCATCATCCAGCCATGAAAAAC	336
GNAT3_8	TTATGGCTCAAACTCTATTGCAG	TGTATGGTCAAAATTCCTTTTTAATG	321
GNAT3_9	TTGCACAGGAACTATGACCACTC	CCAGACTTCACCACTATGCAGTT	346
GNAT3_10	TCAGGTTTTTGAAAGCAAAAGGGA	GCTAGGTACTTCAGCAGAGCATT	263
PLCB2_1	GGGCTGAACCTCCTTGCTAAAT	TCCGAACATGGATATTGGGCATC	349
PLCB2_2	GACCCCACAGTGAAGGATGATG	TGTCTGTGTTGTCCTGATTCTCT	255
PLCB2_3	CATCTCCTAAGGGCAAAGTCAGG	CCTGCCCTATGTTGGATTTCTCA	315
PLCB2_4	CTTTCCACCTGAAGTGGGGTC	CTTCTCGCCAAGCTTACTTCCTC	347
PLCB2_5	CCAACTCTCGCAGCTCCTTC	GGTGTGGAGGAGGCGAAC	324
PLCB2_6	TTTTAAAGGGAAGTGCCTGTGGG	GAAGTCTGTGAAGCTCAAGGAGG	329
PLCB2_7	CTTCTGTGTAGGGAGAGCAGGT	GTTGGGGTGGGATCCAGATG	344
PLCB2_8	CTGGAAGTCTCCCCTGTCCT	CTTGGGTCCTAGGCTGTGTG	315
PLCB2_9	ACTGTTCTGCCCTACTCCTTACA	ACCATGTTCTCACACTGATGCTG	335
PLCB2_10	GAGCTGCCTTGTTTACTTCCTCT	AGAACGAATGCTTCAGCTCTAGG	348
PLCB2_11	TTTCCAGGATATCAGGGGAGGG	AATGGAGGAAATGGAGACTGGG	335
PLCB2_12	TGCATTTCTACCCCCAAATTCTCA	GTTAGGTTCTCCTGGTGCAGATG	337
PLCB2_13	CTTGTTGTAGCTGTCCCTGAGTT	GAGGCATCTCTATAGGCCAGAGG	339
PLCB2_14	CTGACTCCGTGGCTGGATATTTA	TAAGAAGATGCAGTCGGATGAGG	349
PLCB2_15	AGAAGAGGGGAGGGCAAAGAA	GGTGAGTCGGGGAGGACTAT	348
PLCB2_16	ACATACAGCTCTGTCGGCACT	CTGGTAATAAGGAGCTCAGGCTAT	339
PLCB2_17	CAAGTGAACCCTAGCATCCTCTG	ATCACCCTAAGGCCTCATGTTCT	342
PLCB2_18	GAACATGAGGCCTTAGGGTGATT	TGTGTGTATTGAAACTAGGCAGGG	325

PLCB2_19	CTCTAGCTTCAGCCCTCCAAC	GCCCTGAGAATAACACAGACCAT	347
PLCB2_20	GGAACCCCTGTGAAAGCAGAG	CTCATCGACAAGTATGAGCCCAG	327
PLCB2_21	AGAAACCAGACCATGCCTTCAG	GGATATGGACCTGTGCTCTTCTC	325
PLCB2_22	GTAGGGTTTGGCCTTAGCAT	GCAGGGGCAATAAGGTTACTAT	316
PLCB2_23	GGGGTGACCCTGCTTACAAC	TGTGGTTGATGGACTGTGTTCAG	297
PLCB2_24	CATTCCTGGCCAAGTTGCTTTCT	CCTTGACGGGCTGCTTTCT	332
PLCB2_25	TGAGAAAGAAAGGCTTTGGGGAG	CCTGGACTTCCTGAGAGACACA	347
PLCB2_26	GATGTTTGACTAGGGCCAGTACG	TCCATCCTAACCATACCACCAC	328
PLCB2_27	TCAGAAGTCAGGCTTCCCAC	CCATAGGAAGGGAAGTTTGGGTC	343
PLCB2_28	TGTCTGGCCTCAATGATAAGCAG	GAAATGGCATCCCCAATCCTCT	318
PLCB2_29	CATAAACCAATCCTCCCACCCAA	AGAGCAACTAGATTTCTGGAGCA	315
PLCB2E26_1	GGAGCTAAAGGGCGTGGT	GGGGACAGGCCCTGAGA	100
PLCB2E27_1	CAGGATCTCAGGGCCTGTC	GAGTCGGTGAGGGGGTTG	100
RAC1_1	AGGGTACCAATGTGTATGTGGTG	ACAGCAAAACAAATGGTCAAAGA	318
RAC1_2	TCTTGGCACACCTTCTCTAGGAT	TTTACATGGAACCAATCCCACCC	350
RAC1_3	TTGTATGCTTTTGGATCTCTCCG	TTTCATCAAGCAAAACCCCAACC	330
RAC1_4	GTCCAACAAGTCCTTCCCA	AACTAAAAATTCCTAGGCTAAGTG	253
RAC1_5	GGACATCTGTAAAGGAGCGTGTC	TGGTTCCCCAAGAGTACACCAG	337
RAC1_6	GTGGTGTGATCAGAAGAGAGTG	TCTGTAACAAAAAGTTGGCATTGA	328
RAC1E01_1	TGCAGTTTTCCTCAGCTTTG	ACCGCCTACGCGAGACC	100
RALB_1	TAGGTTGAAAGCAAATCGCCTCT	AGCTATTCAGAAGCCACCTCAAC	347
RALB_2	AGTGCCCATATGTGGAATTTTGTT	GAAAAGGCAAAAAGCTTCCTGGG	345
RALB_3	TCACAAAACCAGGGTTCGTTCTT	GTTCCCTAAAACCCAGCAGATGA	326
RALB_4	TCACAGTGTCAGGTTAACAAAAGAA	TTTGAGTGAATGAAGTCGGGGAG	325
